# Risk perception, but also political orientation, modulate behavioral response to COVID-19: A randomized survey experiment

**DOI:** 10.3389/fpsyg.2022.900684

**Published:** 2022-08-17

**Authors:** Fernando Torrente, Daniel Low, Adrian Yoris

**Affiliations:** ^1^Institute of Neuroscience and Public Policy, INECO Foundation, Buenos Aires, Argentina; ^2^Institute of Cognitive and Translational Neurosciences, CONICET, INECO Foundation, Favaloro University, Buenos Aires, Argentina; ^3^Program in Speech and Hearing Bioscience and Technology, Harvard Medical School and Massachusetts Institute of Technology, Boston, MA, United States

**Keywords:** risk perception, protective health behaviors, psychological distress, lockdown, political orientation, anchoring, health psychology, COVID-19

## Abstract

Prior work has shown that accurately perceiving the risk for COVID-19 is associated with higher adherence to protective health behaviors, like face mask use, and more acceptance of governmental restrictive measures such as partial or complete banning of indoor activities and social gatherings. In this study we explored these associations at the beginning of the second wave of COVID-19 in Argentina through a national representative probabilistic survey that evaluated personal and contextual risk perception, self-reported compliance with protective health behaviors, attitude to governmental restrictive measures, and political orientation and psychological distress as potential modulators. Also, going beyond measures of association, here we sought to test whether messages highlighting potential risks increased acceptance of restrictive measures. Three types of messages were randomized to the participants. Two messages conveyed risk-related content (either through emotional arousal or cognitive appraisal) and the third a prosocial, altruistic content. Between March 29th and 30th, 2021, 2,894 participants were recruited (57.57% female). 74.64% of those surveyed evaluated the current health situation as “quite serious” or “very serious” and 62.03% estimated that the situation will be “worse” or “much worse” in the following 3 months. The perception of personal risk and the level of adherence to protective behaviors gradually increased with age. Through a regression model, age, perceived personal risk, and contextual risk appraisal were the variables most significantly associated with protective behaviors. In the case of the acceptance of restrictive measures, political orientation was the most associated variable. We then found messages aimed at increasing risk perception (both emotionally or cognitively focused) had a significantly greater effect on increasing the acceptance of restrictive measures than the prosocial message, mainly for government supporters but also for non-supporters. However, the level of response was also modulated by the political orientation of the participants. We propose a mechanism of “ideological anchoring” to explain that participants were responsive to risk modulation, but within the limits established by their pre-existent political views. We conclude that messages highlighting risk can help reinforce the acceptance of restrictive measures even in the presence of polarized views, but must be calibrated by age and political orientation.

## Introduction

During the last weeks of March 2021, the number of new cases of COVID-19 increased progressively in Argentina, raising the level of alert among experts, government officials, and the general public about the possibility of a second wave of cases in the country. In the weeks that followed, the concerns became reality and the incidence of new cases and mortality surpassed the worst numbers seen during 2020. By March 28th, 2021, vaccination coverage against COVID-19 in Argentina (6.56% of the total population with a single dose and 1.6% with two) was not strong enough to protect against the dissemination of new strains of SARS-CoV-2 and the beginning of winter in the global south. In this context, people’s adherence to individual protection measures, such as the use of face masks, physical distancing, and the avoidance of enclosed social spaces, became essential to prevent the uncontrolled spread of the virus.

Previous knowledge about the adoption of health protection behaviors during pandemics emphasized the role of risk perception in motivating the behavioral response (Bish and Michie, 2010). Perceiving oneself to be more susceptible to contracting the disease and perceiving the disease to be more severe were both associated with taking preventive and avoidant behaviors during pandemics (Floyd et al., 2000; Bish and Michie, 2010). During the COVID-19 pandemic, Bruine de Bruin and Bennett (2020) found a greater reported implementation of protective behaviors to avoid COVID-19 among participants who perceived greater infection risk and greater infection fatality risk. Dryhurst et al. (2020) observed that risk perception was correlated positively and significantly with an index of preventative health behaviors that included washing hands, wearing a face mask, and physical distancing. In a longitudinal study, Schneider et al. (2021) realized that although risk perception varies between different time points, it is consistently associated with the reported adoption of protective health behaviors. Also, gender and age have been found to influence protective health behaviors, with women and older people being more likely to carry out protective health behaviors (Bish and Michie, 2010). Correspondingly, two former studies in Argentina during the COVID-19 pandemic showed that being older was associated with higher risk perception (Torrente et al., 2021, 2022). In addition, there is evidence of different patterns of temporal variation in adherence to protective health behaviors. Low-cost and habituating behaviors like mask-wearing tend to show a monotonic increase in adherence, whereas high-cost behaviors such as physical distancing appear to follow a U-shaped pattern with adherence quickly dropping followed by a small rebound (Petherick et al., 2021).

Meanwhile, risk perception seems to be influenced by multiple different factors. Older age, identifying as female gender (with a binary item), prosocial tendencies, higher self-efficacy, and confidence in science and medicine tend to be associated with a higher perception of risk; in contrast, younger age, identifying as male, and individualistic worldviews are more commonly associated with lower perception of risk (Schneider et al., 2021). Finally, political ideology also plays a role in risk perception, with more conservative citizens being more likely to report lower levels of risk perception (Bruine de Bruin et al., 2020; Schneider et al., 2021).

Together with the reinforcement of individual protective behaviors, the imminence of a second wave rekindled public debate on the need for new restrictive measures on general mobility by the government after a more relaxed period during the previous summer months. During the COVID-19 pandemic’s first wave, Argentina has been considered one of the countries with the highest restrictions [Argentina ranked 14th out of 184 countries on the mean stringency index from January 2020 to April 2020 according to the Oxford Stringency Index, (Torrente et al., 2021)] with a continued strict lockdown of at least 8 months (March 2020 to December 2021). During the first months of the lockdown, adherence was high among Argentines and was associated with the perceived risk (Torrente et al., 2021, 2022). However, psychological distress was also elevated during that period (Torrente et al., 2021, 2022). Furthermore, political ideology influenced citizen support for restrictions in Argentina even more than public health concerns (Freira et al., 2021) as was the case in other countries such as the United States (Grossman et al., 2020; Clinton et al., 2021). Therefore, the announcement of new restrictions could be a driver for greater political polarization with the consequence of less support and erosion of the effectiveness of health measures.

The first aim of this study was to evaluate the population’s risk perception in the face of the second wave of COVID-19 in Argentina and its relationship with the adoption of protective health behaviors and the acceptance of restrictive measures. We also measured the following potential confounds: psychological distress, political orientation of the participants, vaccination status, and sociodemographic characteristics. A national probabilistic representative survey was conducted early on when the incidence of new cases began to increase in the last days of March 2021. We expected that health protective behaviors would be associated with perceived risk. Regarding confounders, we expected that being younger and experiencing greater psychological distress would be associated with less adherence to health protective behaviors. Instead, we anticipated that the acceptance of restrictive measures could also be influenced by political orientation, in addition to risk perception, due to polarization observed during the first wave.

The second aim of this study was to assess the effect of risk-based messages on the acceptance of restrictive directives from the government. Previous research has shown that messages that heighten risk appraisals change intentions and behaviors related to health protection and safety in different domains, such as vaccination, dieting, dental hygiene, sun protection, driving, or medical testing (Sheeran et al., 2014). However, the acceptance of government restrictions is plausibly a different kind of health behavior, more heavily influenced by factors distinct from risk perception, like ideology and trust (Freira et al., 2021). Considering these differences with other health behaviors, it could be of interest to know if acceptance of restrictions could be modulated also by messages that heighten risk appraisals.

To evaluate this, three alternative messages were randomized to the participants in the final section of the survey. The first message was designed as an informational update aimed at increasing risk perception from a cognitive appraisal process. The second message was designed to increase risk perception by triggering emotional arousal. Finally, the third message was conceived as a control message aimed to achieve a prosocial effect, rather than increasing the perception of risk. We hypothesized that the risk-targeted messages, and in particular the emotionally charged message, will have a greater effect than the pro-social message in promoting acceptance of restrictions.

The information obtained could be useful in formulating recommendations to guide public policies based on behavioral and communication factors.

## Materials and methods

### Participants and procedures

We carried out a representative probabilistic population survey at the national level through the Aresco Instant Research Platform.^1^ Data collection took place between 29th and 30th March 2021.

The first part of the survey explored COVID-19 risk perception and protective health behaviors, as well as socio-demographical characteristics of the participants and other variables of interest.

In the final section of the survey, the effect of three types of randomized messages on the intention to accept stricter government restrictive measures against COVID-19 in the next 3 months was evaluated. The three types of messages were based on: (1) increasing perceived risk through an informational update based on comparative epidemiological facts (“cognitive risk enhancement message”); (2) increasing perceived risk through an emotionally loaded description of the new strains of the COVID-19 (“emotional risk enhancement message”); (3) activating prosocial attitudes targeted to take care of more vulnerable people (“prosocial message”).

This project was approved by the Ethics Committee of the Favaloro Foundation.

### Measures

The survey explored the following dimensions:

#### Sociodemographic characteristics

Data were collected on age range, gender, educational level, socioeconomic status, and the region of the country where the participants live.

#### Appraisal of current and future health context

Two questions were included as measures of contextual risk perception (see Supplementary Table 1 in the Supplementary material for a description of the questions). The first question evaluated the participant’s appraisal of the severity of the current health situation of COVID-19 in Argentina, considering the number of new cases, mortality, and ICU beds occupancy. This variable was called “appraisal of current health context” and was ranked along a four points scale from “not serious at all” to “very serious.” The second question assessed the prognosis for the future sanitary situation of COVID-19 during the following 3 months (called “appraisal of future health context,” and ranked in a five points scale from “much better” to “much worse”).

#### Personal risk perception

Risk perception of participants was conceptualized as a multifaceted construct with cognitive and emotional dimensions following the previous literature (Floyd et al., 2000; Bish and Michie, 2010; van der Linden, 2015; Dryhurst et al., 2020). Consequently, for the present study, the perception of personal risk of COVID-19 was evaluated through three questions addressing the perceived severity of the disease by the participants in the event of contracting the COVID-19 virus (perceived severity), the perceived likelihood of being infected by the virus (perceived susceptibility), and the current level of fear of the virus (fear of COVID-19). See Supplementary Table 1 in the Supplementary material for a description of the three corresponding questions. Each question was scored on a four-point scale. For categorical analysis of each dimension, participants who selected the two higher options in the four-point scale were categorized in the “high-perceived risk” group, while those who selected the lower options were categorized in the “low-perceived risk” group. For example, for perceived severity, those participants who selected “severe” or “very severe” were categorized in the “high-perceived severity” group, and those who selected the two remaining options were categorized as the “low-perceived severity” group. The same categorization procedure was used for perceived susceptibility and fear of COVID-19. In addition, for quantitative analysis, an index [personal risk index (PRI)] was calculated as the sum of the scores of the three dimensions.

#### Protective health behaviors

Participants’ self-perceived level of compliance with three types of protective health behaviors (use of a mask, physical distancing, and avoidance of enclosed, non-ventilated places) were surveyed. See Supplementary Table 1 in the Supplementary material for a description of the three questions corresponding to this dimension. The scores of the three individual items were added to calculate a composite index [protective health behavior index (PHBI)]. For categorical analysis, participants who frequently failed to adhere to at least one of the three typified health-protective behaviors were considered as “non-compliant.”

#### Support for restriction measures

The survey included a question that evaluated the participant’s agreement with different levels of hypothetical mobility restrictions as a response to a sustained increase in new cases. The question included five incremental options ranging from “allow all activities” to “total lockdown,” and the participants had to choose the one they considered the more adequate in case of a sustained worsening of the health scenario. See Supplementary Table 1 in the Supplementary material for a description of the question corresponding to this dimension.

#### Psychological distress (patient health questionnaire-4)

The level of psychological distress was assessed using the patient health questionnaire-4 (PHQ-4). The PHQ-4 is an ultra-brief self-report standardized questionnaire that integrates the two-item screening scale for depression PHQ-2 and the two-item screening scale for anxiety GAD-2 (Kroenke et al., 2009). The global score of the PHQ-4 may be considered as a general marker of psychological distress, while a score of 3 or more in either the PHQ-2 or the GAD-2 may be indicative of a possible depressive or anxiety disorder, respectively.

#### Political orientation of the participants

The political orientation of the participants was assessed by asking which candidate they voted for in the previous 2019 presidential elections in Argentina (where the incoming president won with 48% of the votes). For a categorical analysis, the participants were grouped as “government supporters” if they voted for the president of the country at the time of the survey in 2021, and as “opposition supporters” if they had voted for any other candidate.

#### Effect of randomized messages on acceptance of restrictions

Following the randomized messages (see Supplementary Table 2 in the Supplementary material for a description of the three types of messages), a question evaluated the acceptance of potential restriction measures [“Taking into account (content of the message), what level of restrictions would you be willing to accept?”], with four possible answers: (a) “All necessary restrictions,” (b) “Some additional restrictions,” (c) “Only the current restrictions,” (d) “No restrictions at all.” The two lower options in terms of restriction grade (“c” and “d”) were scored with “0,” and the participants who chose these options were categorized as “non-responders.” The participants who chose option “b” were scored with “1” and were categorized as “partial responders.” Finally, option “a” was scored with “2,” and participants who selected it were considered “complete responders.”

### Analysis

For prediction purposes, that is to test how well the three main target variables (PRI, PHB index, and support for restrictions) can be predicted from the remaining variables, we fit Least absolute shrinkage and selection operator (LASSO) multiple regression models for each of the three dependent variables. Our metric of interest is *R*^2^ as a goodness of fit measure. To avoid overfitting (i.e., obtaining a measure that does not hold on new data), we performed stratified 5-fold cross-validation using sklearn’s StratifiedShuffleSplit function (i.e., randomly stratified so the different folds contain similar distributions of variables) with a 80:20 train:test split for each fold to compute the mean out-of-sample (OOS) *R*^2^ and standard deviation across test-set folds. Again, to avoid overfitting, the L1-penalty alpha coefficient was determined through nested cross-validation using 10-fold cross-validation on the training set by testing 30 equally spaced values on a log scale from 0.0001 to 1. Then, the optimal penalty coefficient was used to train a model and predict the test set of each fold to estimate how the model performs on samples it did not use for training (i.e., OOS prediction). To compute coefficients and *p*-values for statistical explanatory inference purposes, we used Python’s statsmodels implementation of LASSO given *p*-values are not provided in the sklearn implementation and conversely, statsmodels does not provide the nested stratified cross-validation procedure we performed for prediction. We use the average L1-penalty derived from the prior cross-validation and fit LASSO on the entire dataset as is customary when computing regression coefficients and *p*-values (Liu and Dobriban, 2019) (i.e., the goal is not prediction but to explain the relationship between variables in this dataset using a null-hypothesis testing approach). To make sure estimates are robust to the penalty used (L1-penalty), we also re-did these analyses with Ridge regression with L2-penalty.

Correlations between measures were carried out by using the Pearson correlation coefficient with Bonferroni correction for multiple comparisons. When analyzing categorical variables, the Pearson chi-square test or the Fisher exact test for 2 × 2 tables were employed.

Regarding the effect of the randomized messages on the acceptance of restrictive measures, due to the characteristics of the dependent variable (an ordinal variable with three categories: non-response, partial response, and total response), an ordinal logistic regression was performed with treatment group as factor. This analysis allowed us to obtain the odds ratio of the effect of the two risk-related messages compared to the pro-social message.

Given political orientation has been shown to influence attitudes toward restrictions in previous literature and it was also the main variable predicting support for restrictions in the LASSO regression analysis, we wanted to ascertain its effect on acceptance of restrictions together with the type of message received. For this reason, we included political orientation in the ordinal regression as a covariate. However, after including the new independent variable, goodness-of-fit testing of the ordinal logistic regression was not adequate. Hence, we changed regression link function to complementary log–log, instead of logit, as the former yielded the best fitting. In the “Results” section we preserved the two ordinal regression analyses, as long as the logit regression allowed us to calculate the odds ratio as a direct measure of the comparative effects of the message, while the complementary log-log regression allowed us to establish whether the message effects were significant after including political orientation as a covariate.

Finally, the odds of eliciting the highest outcome category (“complete response”) was compared between the three types of messages. Odds ratios were obtained for the total sample and for subgroups according to political orientation.

## Results

**FIGURE 1:**
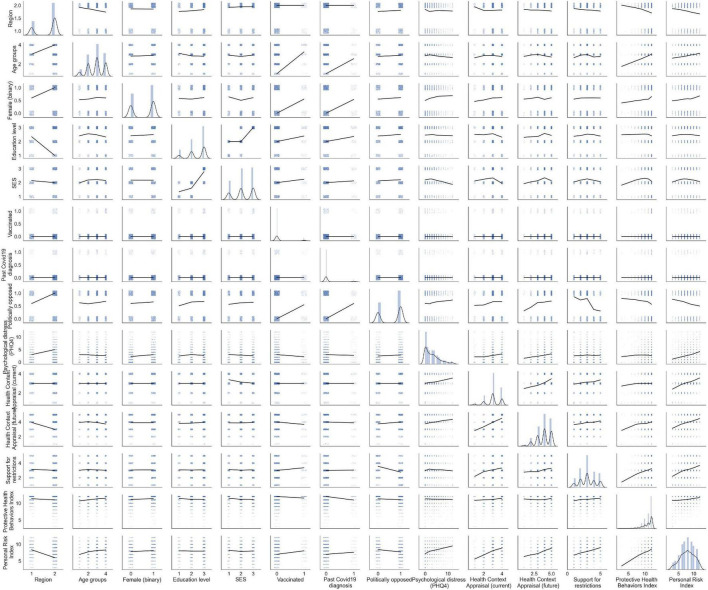
Pairwise scatter plots between variables. Locally weighted linear regression lines are added for illustrative purposes of how the data is distributed, not for statistical inference. The univariate distributions are displayed on the diagonal.

The final sample included 2,894 participants (57.57% female). Table 1 shows descriptive data of the sample, including sociodemographic characteristics, relevant health information, and the political orientation of the participants. At the time of the survey, 6.63% of the sample reported having been previously infected with COVID-19. Regarding the vaccination status, only 7.84% were already vaccinated, while 74.64% of the participants were willing to be vaccinated when available, 14.82% expressed their refusal to be vaccinated, and 2.7% still had doubts. See Figure 1 for a pairwise scatter plot of variables used in the regression analyses.

**TABLE 1:** Sociodemographic and descriptive data of the sample (*n* = 2,894).

	*n*	%
**Gender**		
Female	1,666	57.57
Male	1,228	42.43
**Age**		
Up to 29	240	8.29
30–49	749	25.88
50–65	1,109	38.32
66+	796	27.51
**Socio-economic Status**		
Low/medium low	597	20.63
Medium	985	34.04
Medium high/high	1,019	35.21
Do not report	293	10.12
**Education level**		
Primary	424	14.65
Secondary	922	31.86
University	1,548	53.49
**Region**		
AMBA	875	30.23
Inside the country	2,019	69.77
**Infected with COVID-19 before**		
No	2,702	93.37
Yes	192	6.63
**Vaccination status**		
Yes	227	7.84
Willing to be vaccinated	2,160	74.64
Not willing to be vaccinated	429	14.82
Don’t know yet	78	2.70
**Political orientation**		
Government supporters	1,126	38.91
Opposition supporters	1,768	61.09

AMBA, Metropolitan Area of Buenos Aires.

### Appraisal of current and future health context

Most of the participants (74.64%) appraised the current health context at the moment of the survey as “quite serious” or “very serious” (see Supplementary Table 3 in the Supplementary material). Appraisal of current health context was uniform across age groups.

Regarding the evolution of the health context for the following 3 months after the survey, 62.11% of the participants estimated that the situation would be “worse” or “much worse.” There were significant differences in the estimations made by participants of different age groups (χ^2^ = 47.53; *p* < 0.001; see Supplementary Table 3). The older group (+66) was the less pessimistic about the future, with the lower rate of participants (55%) imagining a worsening scenario.

### Perception of personal risk

Perceived personal risk associated with COVID-19 was evaluated in three dimensions: perceived severity, perceived susceptibility, and fear to COVID-19 (see Supplementary Figure 1; Supplementary Table 4 in the Supplementary material). For perceived severity, 41.84% of the participants stated that the disease would be “very severe” or “severe” in case of infection (high-perceived severity group), while 48.13% perceived a lower severity. Perception of severity increased steadily across age groups (see Supplementary Figure 1A). The differences in the distribution of participants in high and low severity categories across age groups were significant (χ^2^ = 63.14; *p* < 0.001; see Supplementary Table 5 in the Supplementary material).

Considering perceived susceptibility, 40.32% of the participants considered “very” or “quite” likely to be infected with COVID-19, while 47.30% perceived themselves as “a little or nothing at all” susceptible to be infected. There were also age-related differences in this dimension (χ^2^ = 11.32; *p* = 0.01; see Supplementary Figure 1B; Supplementary Table 5), with the group of 30–49 years old having the higher percentage of participants in the high-susceptibility group (44.86%).

Regarding the third component of personal risk appraisal, 78.23% of the participants expressed fear of COVID-19. This dimension also increased steadily across age groups (χ^2^ = 35.57; *p* < 0.001; see Supplementary Figure 1C; Supplementary Table 5 in the Supplementary material).

### Protective health behaviors

**FIGURE 2:**
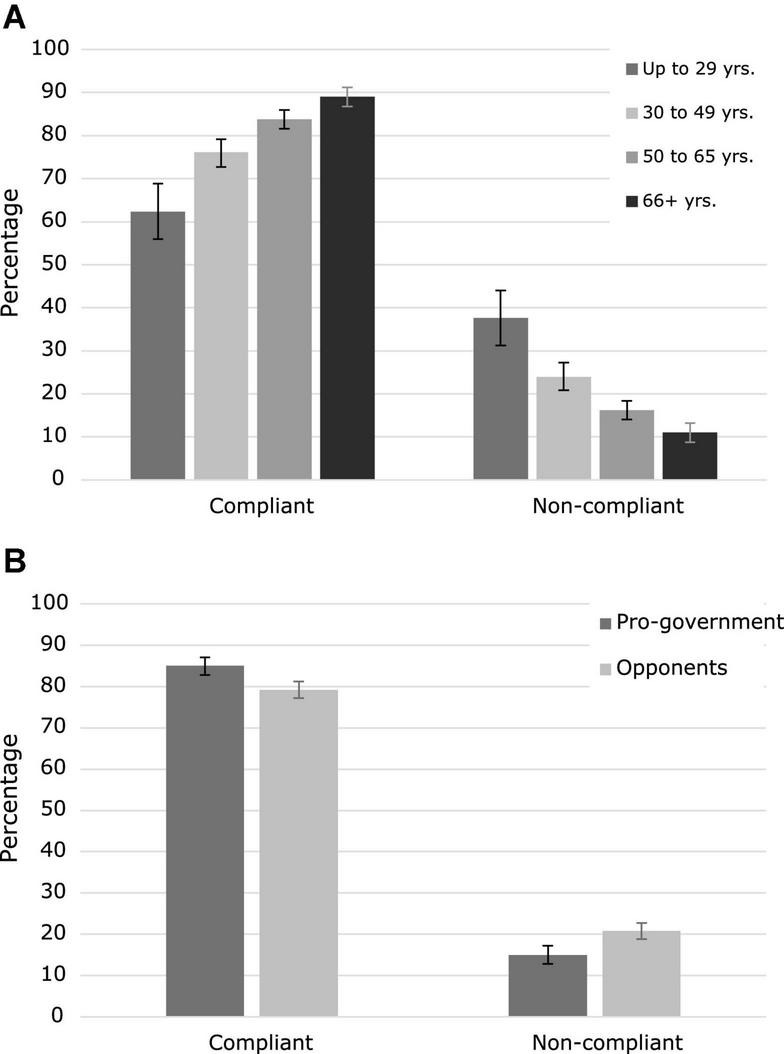
Compliance with protective health behaviors. **(A)** Percentage of participants compliant with protective health behaviors according to age group. **(B)** Percentage of participants compliant with protective health behaviors according to political orientation. Error bars represent bootstrapped 95% CI.

Table 2 shows the distribution of the participants according to their compliance with protective health behaviors. The majority of the participants (81.45%) perceived themselves as globally compliant with the three protective behaviors against COVID-19. Segmented by the three specific behaviors assessed, compliance was greater for use of masks (83.28% of the total sample) than for physical distancing (68%) and avoidance of indoor closed places (65.27%) (see Supplementary Table 6 in the Supplementary material). Non-compliance increases as age descends (see Table 2 and Figure 2). The differences in the distribution were statistically significant (χ^2^ = 103.01; *p* < 0.001). The rate of non-compliance in the age group under 29 years old was 37.61%, doubling the percentage of the total sample (18.56%). Rates on non-compliance also differed between political orientation groups (Fisher exact test, *p* < 0.001), even if both supporters of government and opposers were mostly compliant (85.03% vs. 79.15%, respectively; see Table 2 and Figure 2).

**TABLE 2:** Compliance with protective health behaviors.

				Bootstrapped 95% CI for%*	
				
	Compliant	*n*	%	Lower	Upper
**Total sample**					
	Yes	2,300	81.44	80.06	82.86
	No	524	18.56	17.14	19.94
**By age (years)^1^**					
Up to 29	Yes	146	62.39	55.98	68.80
	No	88	37.61	31.20	44.02
30–49	Yes	558	76.13	72.72	79.13
	No	175	23.87	20.87	27.28
50–65	Yes	908	83.76	81.64	85.98
	No	176	16.24	14.02	18.36
66+	Yes	688	89.00	86.80	91.20
	No	85	11.00	8.80	13.20
**By political orientation^2^**					
Pro-G	Yes	937	85.03	82.76	87.11
	No	165	14.97	12.89	17.24
Opp	Yes	1,363	79.15	77.24	81.18
	No	359	20.85	18.82	22.76

Pro-G, government supporters; Opp, opposition supporters.

*Bootstrapped 95% CI were calculated from 1,000 samples.

^1^χ^2^ = 103.01; p < 0.001.

^2^Fisher’s exact test, p < 0.001.

### Support for restriction measures

**FIGURE 3:**
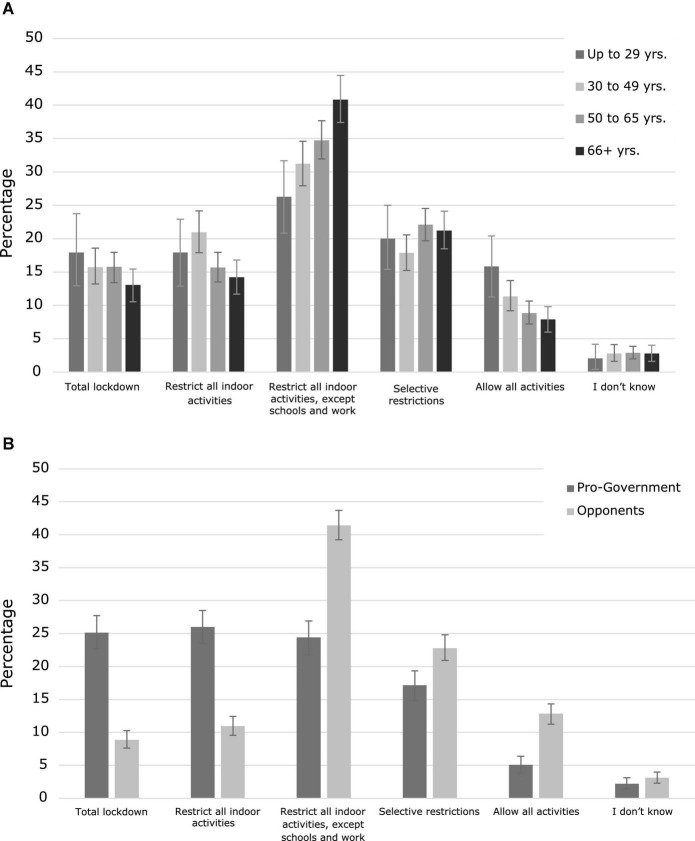
Support for government restrictive measures. **(A)** Percentage of participants supporting the different levels of restriction according to age groups. **(B)** Percentage of participants supporting the different levels of restriction according to political orientation. Error bars represent bootstrapped 95% CI.

Table 3 shows the participants’ support for different levels of hypothetical restrictive measures to be adopted in the case of a sustained increase of COVID-19 new cases. Most of the participants (67%) considered it convenient to adopt more restrictions as a response to a worsening of the sanitary situation. However, the majority of them showed a preference for middle-intensity measures over measures of total restriction. At the same time, a relevant minority of 30% were against increasing restrictive measures. This relative distribution is maintained when the results are disaggregated by age and political orientation, although government supporters tend to prefer more restrictive measures (see Table 3 and Figure 3).

**TABLE 3:** Participants support for government restrictive measures.

	Level of restriction supported	*n*	%	Bootstrapped 95% CI for %*	
				
				Lower	Upper
Total sample	Total lockdown	440	15.20	13.96	16.55
	Restrict all indoor activities	487	16.83	15.45	18.24
	Restrict all indoor activities, except schools and work	1,007	34.80	33.10	36.66
	Selective restrictions^1^	596	20.59	19.07	21.98
	Allow all activities	284	9.1	8.74	10.88
	I don’t know	80	2.76	2.21	3.39
By age (years)^2^					
Up to 29	Total lockdown	43	17.92	12.93	23.75
	Restrict all indoor activities	43	17.92	12.92	22.92
	Restrict all indoor activities, except schools and work	63	26.25	20.84	31.67
	Selective restrictions	48	20.00	15.42	25.00
	Allow all activities	38	15.83	11.26	20,42
	I don’t know	5	2.08	0.42	4.16
30–49	Total lockdown	118	15.75	13.22	18.56
	Restrict all indoor activities	157	20.96	17,89	24.17
	Restrict all indoor activities, except schools and work	234	31.24	27.90	34.58
	Selective restrictions	134	17.89	15.22	20,56
	Allow all activities	85	11.35	9.21	13.75
	I don’t know	21	2.80	1.60	4,14
50–65	Total lockdown	175	15.78	13.44	17.94
	Restrict all indoor activities	174	15.69	13.53	17.94
	Restrict all indoor activities, except schools and work	385	34.72	31.92	37.69
	Selective restrictions	245	22.09	19.66	24.53
	Allow all activities	98	8.84	7.21	10.64
	I don’t know	32	2.89	1.98	3.88
66+	Total lockdown	104	13.07	10.56	15.45
	Restrict all indoor activities	113	14.20	11.68	16.83
	Restrict all indoor activities, except schools and work	325	40.83	37.44	44.47
	Selective restrictions	169	21.23	18.47	24.12
	Allow all activities	63	7.91	6.03	9.80
	I don’t know	22	2.76	1.64	4.02
By political orientation^3^					
Pro-G	Total lockdown	283	25.13	22.74	27.71
	Restrict all indoor activities	293	26.02	23.53	28.51
	Restrict all indoor activities, except schools and work	275	24.42	21.76	26.91
	Selective restrictions^1^	193	17.14	14.83	19.36
	Allow all activities	57	5.06	3.82	6.39
	I don’t know	25	2.22	1.42	3.11
Opp	Total lockdown	157	8.88	7.64	10.29
	Restrict all indoor activities	194	10.97	9.56	12.44
	Restrict all indoor activities, except schools and work	732	41.40	39.25	43.72
	Selective restrictions^1^	403	22.79	20.93	24.83
	Allow all activities	227	12.84	11.26	14.31
	I don’t know	55	3.11	2.32	3.96

Pro-G, government supporters; Opp, opposition supporters.

*Bootstrapped 95% CI were calculated from 1,000 samples.

^1^Selective restrictions: massive events, nightclubs, indoor meetings with numerous people, restricted number of people indoors, etc. This option reflects the measures in force at the time of the survey.

^2^χ^2^ = 51.13; p < 0.001.

^3^χ^2^ = 324.14; p < 0.001.

### Psychological distress

Regarding depressive symptoms, 25% of the participants showed scores of 3 or more on the PHQ-2, which is the cut-off point suggested to identify people who are likely to be depressed. For anxiety symptoms, 22.6% of the sample scored 3 or more in the GAD-2, indicating a possible anxiety disorder. There were no differences between age groups in PHQ-4 (*p* = 0.261), PHQ-2 (*p* = 0.126), or GAD-2 (*p* = 0.748) according to Kruskal–Wallis test.

### Relationship between risk perceptions, protective behaviors, support for restrictions, and psychological distress measures

Table 4 presents the correlations between PRI, contextual risk appraisals, PHBI, support for restrictive measures, and psychological distress (PHQ-4). Positive significant correlations were found between PRI and the measures of present and future contextual risk appraisal (*r* = 0.330, *p* < 0.001 and *r* = 0.283, *p* < 0.001, respectively), between the PRI and the PHBI (*r* = 0.290, *p* < 0.001), and between the PRI and support for restriction measures (*r* = 0.262, *p* < 0.001). PHQ-4 was positively correlated with PRI (*r* = 0.232, *p* < 0.001), but not with PHBI or support for restrictions.

**TABLE 4:** Pearson’s correlations between risk perceptions, protective behaviors, support for restrictions, and psychological distress measures.

Variable	1	2	3	4	5	6
1. Personal risk index	–					
2. PHB index	0.290*, *p* < 0.001	–				
3. Health context appraisal (current)	0.330*, *p* < 0.001	0.220*, *p* < 0.001	–			
4. Health context appraisal (future)	0.283*, *p* < 0.001	0.139*, *p* < 0.001	0.373*, *p* < 0.001	–		
5. Support for restrictions	0.262*, *p* < 0.001	0.211*, *p* < 0.001	0.179*, *p* < 0.001	0.136*, *p* < 0.001	–	
6. PHQ-4	0.232*, *p* < 0.001	−0.032, *p* = 0.115	0.127*, *p* < 0.001	0.147*, *p* < 0.001	0.001, *p* = 0.947	–

PHB, protective health behaviors; PHQ-4, patient health questionnaire-4.

*Flagged correlations are significant after Bonferroni correction for multiple comparisons (corrected α = 0.003).

### Regression models

Table 5 presents the results of LASSO regression models predicting Support for restriction, PRI, and PHBI measures. Supplementary Table 7 in the Supplementary material presents results for Ridge regression, which are almost identical to the LASSO model results.

**TABLE 5:** Predictive performance and coefficients for LASSO regression.

	Personal risk index OOS *R*^2^ = 0.21 (0.03)			Protective health behaviors index OOS *R*^2^ = 0.17 (0.02)			Support for restrictions OOS *R*^2^ = 0.15 (0.02)		
			
	Covariate	Coef.	*p*	Covariate	Coef.	*p*	Covariate	Coef.	*p*
1	Health context appraisal (current)	0.38***	0.001	Age groups	0.29***	0.001	Politically opposed	−0.32***	0.001
2	Psychological distress (PHQ4)	0.34***	0.001	Personal risk index	0.21***	0.001	Personal risk index	0.15***	0.001
3	Health context appraisal (future)	0.3***	0.001	Health context appraisal (current)	0.19***	0.001	Protective health behaviors index	0.14***	0.001
4	Protective health behaviors index	0.27***	0.001	Support for restrictions	0.17***	0.001	Health context appraisal (current)	0.12***	0.001
5	Support for restrictions	0.25***	0.001	Region	−0.12***	0.001	Health context appraisal (future)	0.09***	0.001
6	Politically opposed	−0.25***	0.001	Female (binary)	0.11***	0.001	Vaccinated	0.06**	0.002
7	Age groups	0.25***	0.001	Psychological distress (PHQ4)	−0.1***	0.001	Age groups	−0.05*	0.02
8	Region	−0.08*	0.017	Politically opposed	−0.1***	0.001	Education level	0.04*	0.049
9	Female (binary)	0.04	0.196	Health context appraisal (future)	0.1***	0.001	Psychological distress (PHQ4)	−0.03	0.178
10	Education level	0.03	0.482	Vaccinated	0.08***	0.001	Past COVID-19 diagnosis	0	–
11	Vaccinated	−0.03	0.392	Past COVID-19 diagnosis	−0.04	0.105	SES	0	–
12	Past COVID-19 diagnosis	−0.03	0.395	SES	−0.04	0.314	Female (binary)	0	–
13	SES	0.01	0.77	Education level	−0.01	0.818	Region	0	–

OOS R^2^ is out-of-sample prediction mean (and standard deviation) across five test sets using 5-fold cross-validation. Variables are ranked by their absolute coefficient values. The standardized coefficients represent how many standard deviations a dependent variable will change per standard deviation increase in the covariate (e.g., 1 SD increase in being politically opposed is associated with 0.32 decrease in Support for restrictions, if all other covariates are fixed). Stronger associations have higher absolute coefficient values. Positive coefficients make it more likely that the dependent variable will increase; negative coefficients make it more likely that the dependent variable will decrease. Coefficients closer to zero are not associated to the dependent variables.

LASSO, least absolute shrinkage and selection operator; OOS, out-of-sample; Coef., coefficient; p, p-value (unspecified for coefficients equal to zero); PHQ-4, patient health questionnaire-4; SES, socio-economic status.

*** = P-value ≤ 0.001; ** = P-value ≤ 0.01; * = P-value ≤ 0.05.

The variables most significantly associated to PRI were current health context appraisal, followed by psychological distress, future health context appraisal, PHBI, support for restriction, being politically opposed (negatively), and age. Together these variables accounted for 21% of the variance of PRI.

The variables most significantly associated to PHBI were age, followed by PRI, current health context appraisal, and support for restrictions. The model accounted for 17% of the variance of PHBI.

Finally, the variables most significantly associated to Supporting restriction measures was being politically opposed followed by PRI and PHBI. The model accounted for 15% of the variance of the dependent variable.

### Effect of risk-related messages

**FIGURE 4:**
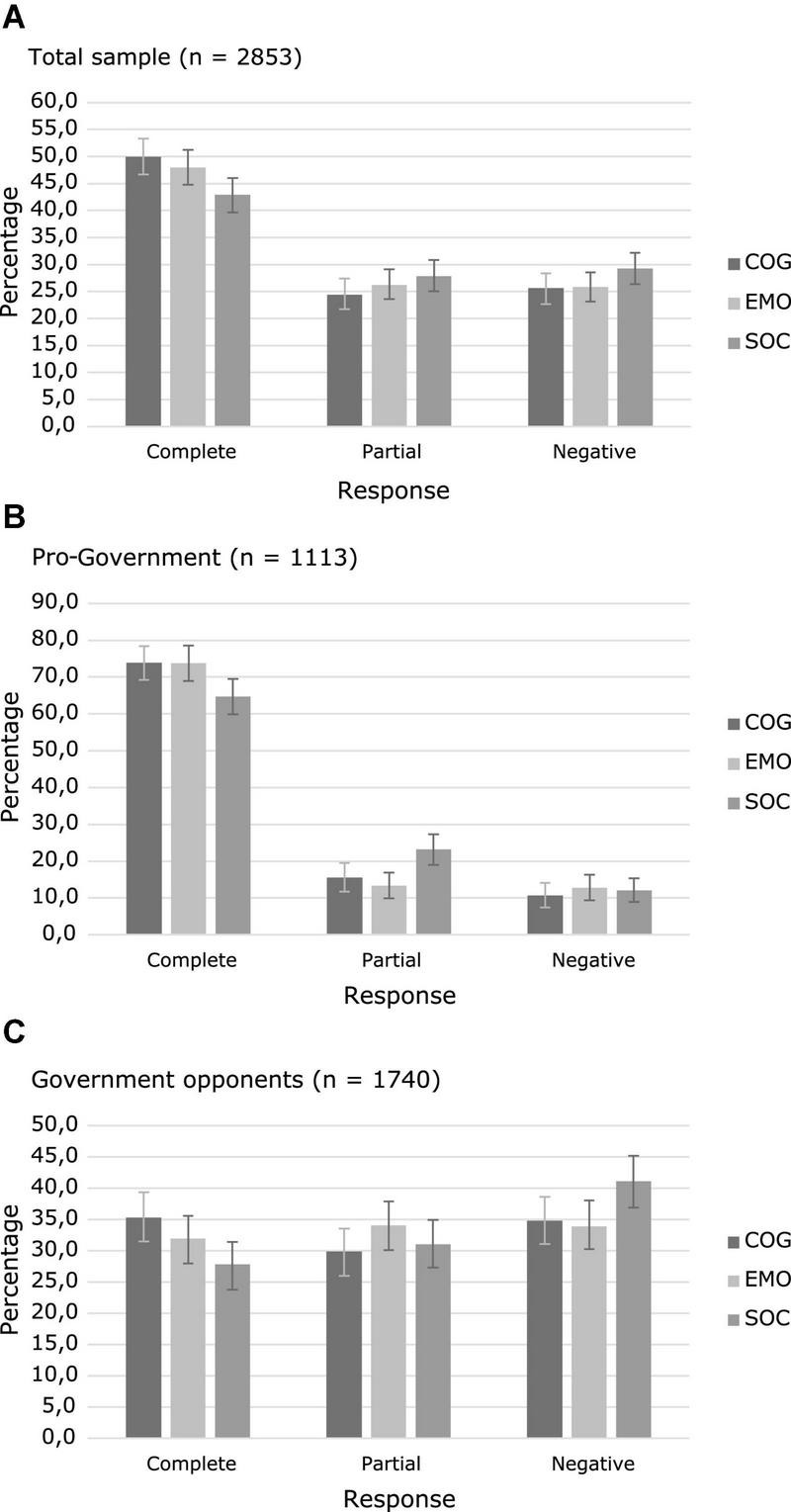
Effect of messages on restrictions acceptance. **(A)** Participants’ responses to the three types of messages in the total sample. **(B)** Participants’ responses to the three types of messages among the government supporters. **(C)** Participants’ responses to the three types of messages among the opposition supporters. Error bars represent bootstrapped 95% CI (COG, cognitive risk message; EMO, emotional risk message; SOC, pro-social message; Complete response: “Support all necessary restrictions”; Partial response: “Support some additional restrictions”; Negative response: “Support only the current restrictions” or “No restrictions at all”).

Table 6 and Figure 4 presents the participants responses to the three randomized messages (see also Supplementary Figure 2 in the Supplementary material).

**TABLE 6:** Outcome of messages by category of response.

	Message	Response	*n*	%	Bootstrapped 95% CI for%*	
					
					Lower	Upper
Total Sample	COG	Complete^1^	458	49.95	46.7	53.3
		Partial^2^	224	24.43	21.7	27.4
		Negative^3^	235	25.63	22.7	28.4
	EMO	Complete	469	47.96	44.8	51.2
		Partial	256	26.18	23.6	29.1
		Negative	253	25.87	23.1	28.5
	SOC	Complete	411	42.90	39.7	46.0
		Partial	267	27.87	25.1	30.8
		Negative	280	29.23	26.4	32.2
Pro-G	COG	Complete	257	73.85	69.25	78.45
		Partial	54	15.52	11.78	19.53
		Negative	37	10.63	7.47	14.08
	EMO	Complete	276	73.80	68.98	78.61
		Partial	50	13.37	9.89	16.84
		Negative	48	12.83	9.36	16.31
	SOC	Complete	253	64.71	59.85	69.57
		Partial	91	23.27	18.93	27.37
		Negative	47	12.02	8.95	15.35
Opp	COG	Complete	201	35.33	31.46	39.37
		Partial	170	29.88	26.01	33.56
		Negative	198	34.80	31.11	38.66
	EMO	Complete	193	31.95	27.98	35.60
		Partial	206	34.11	30.13	37.91
		Negative	205	33.94	30.30	38.08
	SOC	Complete	158	27.87	23.81	31.39
		Partial	176	31.04	27.34	34.92
		Negative	233	41.09	36.87	45.15

COG, cognitive risk message; EMO, emotional risk message; SOC, pro-social message; Pro-G, government supporters; Opp, opposition supporters.

*Bootstrapped 95% CI were calculated from 1,000 samples.

^1^Complete response: “Support all necessary restrictions”.

^2^Partial response: “Support some additional restrictions”.

^3^Negative response: “Support only the current restrictions” or “No restrictions at all”.

An ordinal logistic regression was performed to evaluate the effect of the type of message on acceptance of government restrictions. Results showed that the final model was significant (χ^2^ = 9.038, *p* = 0.011) and the assumption of proportional odds was met (*p* = 0.527). The obtained estimates indicated that the two risk-related messages outperformed the pro-social message. Participants that received the cognitive risk enhancement were significantly more likely to endorse a higher level of acceptance of restrictions than those who received the pro-social message (*B* = 0.246, SE = 0.086, Wald = 8.115, *p* = 0.004, OR = 1.28, 95% CI = 1.080–1.515). The effect of the emotional risk enhancement messages was also significant (*B* = 0.189, SE = 0.085, Wald = 4.976, *p* = 0.026, OR = 1.21, 95% CI = 1.024–1.426; see Supplementary Table 8 in the Supplementary material for the regression tables).

**FIGURE 5:**
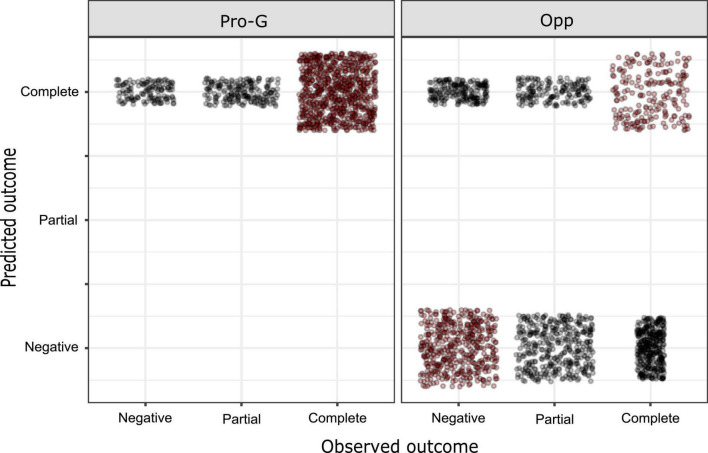
Predicted vs. observed outcomes in ordinal regression. Red dots correspond to matched cases (Pro-G, government supporters; Opp, opposition supporters).

When political orientation was introduced as a covariate, a regression model with complementary log-log link function showed the best fit and was found to be significant (χ^2^ = 454.560, *p* < 0.001). Both the effects of cognitive risk messages (*B* = 0.242, SE = 0.064, Wald = 9.008, *p* = 0.004) and emotional risk messages (*B* = 0.187, SE = 0.062, Wald = 14.270, *p* < 0.001) remained significant. Political orientation also showed a significant effect on the result (*B* = 1.212, SE = 0.063, Wald = 369.267, *p* < 0.001), with government supporters more likely to support a higher level of restrictions (see Figure 5 and Supplementary Table 9 in the Supplementary material for additional regression data).

Finally, Table 7 presents the odds ratios that compare the effect of the three types of messages in eliciting the highest response category (“complete response”). Results are displayed for the total sample and for subgroups according to political orientation. The effects of the two risk-based messages were superior to the pro-social message in the total sample and government supporters subgroups. In the opponents to the government subgroup, only the cognitive risk enhancement message was superior to the pro-social message.

**TABLE 7:** Comparative effect of messages in eliciting the highest response category (complete response).

			95% CI		
			
		OR	Lower	Upper	*p* *
Total sample	COG vs. SOC	1.328	1.107	1.593	0.003
	EMO vs. SOC	1.226	1.025	1.467	0.028
	COG vs EMO	1.083	0.904	1.297	0.408
Pro-G	COG vs. SOC	1.54	1.123	2.114	0.008
	EMO vs. SOC	1.536	1.127	2.095	0.008
	COG vs. EMO	1.003	0.719	1.398	1
Opp	COG vs. SOC	1.414	1.099	1.818	0.007
	EMO vs. SOC	1.216	0.946	1.562	0.142
	COG vs. EMO	1.163	0.913	1.482	0.24

COG, cognitive risk message; EMO, emotional risk message; SOC, pro-social message; Pro-G, government supporters; Opp, opposition supporters; OR, odds ratio.

*Fisher’s exact test.

## Discussion

The first aim of this study was to assess risk perception right before the second wave of COVID-19 in Argentina, and its relationship with protective health behaviors and other associated variables. As a second aim, we explored the effect of risk-related messages on the willingness to accept future sanitary restrictions. For these purposes, a national representative probabilistic survey was carried out.

We evaluated participant’s level of awareness about the worsening of the health situation after a few months of greater relief in Argentina. We found that most of the participants perceived the current and upcoming future health situations as risky. Thus, most people were well-tuned with the objective sanitary scenario. In the regression model, the appraisal of the current and future health contexts were positively associated with personal risk perception and the adoption of protective health behaviors, especially the current health context appraisal. As expected, the awareness about the increase in the rate of new cases and the expectation of a worsening in the curve may have augmented the level of personal risk in the population. Psychological distress was another important variable for predicting personal risk and may reflect the affective dimension of risk perception (Slovic et al., 2005), arising from a bidirectional process where awareness of the worsening of the situation activated negative feelings associated with emotional experiences during the first wave (Torrente et al., 2021, 2022), which in turn reinforced the cognitive component of risk perception.

The perception of personal risk was not homogeneous, being lower for younger people and increasing gradually with age. Even if younger groups had a lower global perceived risk, as revealed by the differences in PRI, differences in risk perception were more noticeable in perceived severity and fear to COVID-19. Previous information and personal experiences during the first wave about the higher lethality and severity of COVID-19 disease in older people may have influenced the tempered perception of risk in the younger population.

Most of the participants (81.45%) perceived themselves as compliant with the protective health behaviors; however, older age groups tended to comply more. Participants who failed to comply with protective behaviors perceived a lower risk and had more mood symptoms. A negative mood may contribute to non-compliance by increased fatigue, social isolation, impaired cognitive functioning, or helplessness (DiMatteo et al., 2000; Wing et al., 2002), but it is also possible going against regulations and social practices may decrease mood.

In the regression analysis, the perceptions of personal risk and contextual risk appraisal were both associated with greater compliance with protective behaviors, and together with age, they were the most important variables to explain protective behaviors. These findings confirmed the hypothesis derived from classic models of health psychology about the relationship between risk perception and adoption of protective health behaviors (Floyd et al., 2000; Bish and Michie, 2010), and were consistent with previous studies during the COVID-19 pandemic in different countries (Bruine de Bruin and Bennett, 2020; Ning et al., 2020; Schneider et al., 2021).

Up to this point, the expectations of a “rational” actor in the health domain were met for most people: participants perceived the external health situation in a realistic way, configured their personal risk based on prior information on susceptibility and severity, cognition and emotion were aligned, and then they adopted protective measures according to the level of perceived risk.

However, a factor outside the health domain also seems to influence perceived risk and health behaviors. In the present study, we confirmed the modulating role of political orientation on risk perception observed in previous studies during the COVID-19 pandemic (Bruine de Bruin et al., 2020; Barrios and Hochberg, 2021). Citizens identified as not-supporters of the current government were more likely to report lower levels of risk perception. Being opposed to the government was also associated with a higher rate of non-compliance with protective health behaviors. However, the differences between groups were small (∼5%) and the political orientation was not found to be an important predictor of individual protective behaviors in regression analysis.

Instead, the modulating effect of political orientation was especially evident in the support for restrictive measures: it was the variable that was most associated to the endorsement of restrictive measures in the regression analysis, over other health and demographic variables. This result is consistent with a previous report in Argentina (Freira et al., 2021).

Why was support for restriction measures so influenced by political orientation, to a much greater extent than other health protective behaviors? The long period of restrictions adopted by the government of Argentina in response to the first wave of COVID-19 ignited a strong political debate because, unlike individual protective behaviors such as wearing a mask or maintaining physical distancing, the adoption of restrictive measures entailed high economical and emotional costs (Deb et al., 2022; Torrente et al., 2022). This debate resulted in a partisan polarization between government supporters and opponents that may have influenced the opinions about the best way of coping with the second wave. Once the issue of restrictions became a subject of partisan discussion, the evaluation of their convenience in cost-benefit terms could have been affected by the action of politically motivated reasoning (Kahan, 2016). This term refers to the selective processing of information about a hot political topic to make it consistent with the beliefs of the affinity group. The goal of this type of reasoning is to maintain group identity rather than achieve the truth. Thus, under this mechanism, political orientation may be a better predictor of an individual’s choice than other factual considerations, such as actual health risks.

Nevertheless, it should be noted that in our study the endorsement of restrictive measures does not appear to be completely polarized or irrationally detached from public health considerations. Regardless of the political orientation, there is a preference in most of the participants for the adoption of active measures over the maintenance of the “*status quo*” or the relaxation of restrictive measures, since 67% of the participants consider it necessary to adopt more restrictive measures than those in force at the moment of the survey, although with a preference for measures of intermediate intensity. These preferences for active but moderate measures suggest an “intelligent” decision process, rather than an automatic polarized alignment. Consequently, in many cases, the influence of political orientation may be a matter of degrees, rather than a dichotomy between extreme positions.

Finally, risk-based messages showed a significantly greater effect than prosocial messages in the intention to adopt stricter restriction measures. The first risk message was designed to elicit cognitive appraisal and make an informative update comparing the rates of infection and mortality the days before the survey with the rates of the first wave. The second risk message was intended to provoke emotional arousal by highlighting the more aggressive features of the new strains of the virus. The two risk messages worked better than a prosocial message reinforcing the importance of adopting restrictions for helping the more vulnerable population. These results showed that participants are in general permeable to interventions that modulate risk perception as previous studies on the matter have shown (Sheeran et al., 2014). Even if the perceived risk was basically different in the groups with different political orientations, risk-related messages were effective in increasing acceptance of restrictions after taking political orientation into account. In particular, the message with cognitive information worked better than the pro-social message in eliciting a full response in government opposers. Thus, the politically biased perception was not completely immune to new information related to health, although the positive response rate to risk-related messages was considerably lower for opponents of the government.

Taking into account the results of the survey and the effects of the messages as a whole, we can affirm that both health-related and health-unrelated variables influence risk perception and the adoption of health protective measures. A matter of further study would be to understand how the two types of information interact. One possible account comes from the application of the anchoring and adjustment heuristic (Tversky and Kahneman, 1974). A hypothetical mechanistic interpretation of the findings of the current study based on that heuristic is that political orientation sets an anchor that establishes the initial parameter from which the risk estimation is made. The opinions of political leaders, partisan slogans, biased information from the media, social networks, or social groups that function as “echo chambers,” may provide a reference to adopting a rough primary position (“this is serious” vs. “this is not serious”) (Ajzenman et al., 2020; Grossman et al., 2020). Then, the evaluation of risk starts from this anchor by making an adjustment after considering the current information on the health situation. The information about infection and mortality rates, along with personal experiences (personal contagion or from close people) may serve as corrective cues to calibrate the initial anchor. As we observed, most people were realistically aware of the health context. Individual risk estimations, such as perceived susceptibility and severity, are also considered for the adjustment but this update process could result in insufficient adjustment, an effect known as anchoring bias, which refers to the tendency to assimilate the final judgment toward the starting point or values close to it (Tversky and Kahneman, 1974; Furnham and Boo, 2011). The anchoring bias has been shown to affect communications about health risks (Senay and Kaphingst, 2009) and in our case, it may help to explain why in presence of factual information, health perceptions may be tied to the initial ideologically biased estimations.

Due to the action of the anchoring bias and the changes in health conditions themselves, it is conceivable that the adjustment process needs to be repeatedly updated at different times of the pandemic. Also, as shown in the experimental part of this study, the messages that increase the sense of risk through health-related information may serve as tools for adjusting the initial highly biased estimations, but the adjustment will be probably still constrained within the surroundings of the basal estimation. The right messages may reduce the bias, but will not make pre-existing differences between groups attributable to the ideologic anchoring disappear. This two-steps risk estimation process based on anchoring and adjustment heuristic could account for the influence of both non-health and health variables on risk estimation and the adoption of protective measures. Future experimental studies could test this.

From the pragmatic perspective, and even within the limits created by this sort of ideologic anchoring, we have shown that health protective behaviors are linked to risk perception, and that risk perception may be enhanced by messages that operate at both informational and emotional levels of the risk perception process. Timely and accurate health information held by neutral non-partisan sources may result in desirable changes in the intention to support and to adopt protective behaviors, even if limited in magnitude.

The present study has several limitations. First, the survey is based on self-report methods and therefore carries the biases typical of this type of subjective measurement. Second, our analyses are not intended to estimate the causal relationships between the variables assessed in the first part of the survey before the messaging. Third, the amount of variance explained in the three regression analyses was small, leaving a lot of room for unrecognized variables in explaining the phenomena studied. Fourth, the effect of the messages was measured as self-reported intentions, not as actual behaviors, and it is known that intentions do not always materialize as anticipated.

As a matter of future studies, other factors not included in this report may have influenced the observed results and may help to understand the unexplained variance of the regression analyses. Non-health variables, such as institutional trust (Han et al., 2021; Pagliaro et al., 2021) or economic burden (Barnett-Howell et al., 2021), can affect health outcomes and behaviors. Over time, fatigue and habit formation may also have played a role, as some measures are more expensive to maintain in the long term, such as physical distancing or activity restrictions, while others, such as mask-wearing, could be easier to sustain (Petherick et al., 2021). Additionally, having adequate scientific knowledge about COVID-19 and susceptibility to misinformation may affect perceived risk and health behaviors (Roozenbeek et al., 2020). Finally, although we focused on contextual variables, it is possible to think that, to some extent, some behaviors could have been influenced by dispositional factors, such as personality traits or beliefs and attitudes formed at the beginning of the pandemic.

In conclusion, the model that emerges from this study is that of health behavior governed by risk perceptions, with both elements moderated by political ideology. Risk perceptions respond to contextual factors and to information related to health and medical issues, but political orientation also is associated with the perceptions and behaviors toward health, especially in the more polarizing topics. Despite this, political ideology does not seem to be completely decoupled from health information, since even within the parameters of each group, certain rational relationships between the variables linked to health are maintained. At the individual level, those who perceive greater risk take more care of themselves. Most people prefer selective and intelligent measures, over the most extreme. Also, the modulation of risk through health-related information seems to exert an effect that is not completely neutralized by the influence of political ideology.

## Data availability statement

The datasets presented in this study can be found in online repositories. The names of the repository/repositories and accession number(s) can be found below: github.com/danielmlow/covid19_risk.

## Ethics statement

The studies involving human participants were reviewed and approved by Comité de Ética Independiente de la Fundación Favaloro, Buenos Aires, Argentina. Written informed consent for participation was not required for this study in accordance with the national legislation and the institutional requirements.

## Author contributions

FT and AY conceived the design, performed the data analysis, and drafted the manuscript. DL contributed to the data analysis and writing. All authors provided feedback for the discussion.

## Footnotes

1
http://aresco-ir.com/about_en.php

## References

[B1] Ajzenman N. Cavalcanti T. Da Mata D. (2020). *More than words: Leaders’ speech and risky behavior during a pandemic*. Available online at: 10.2139/ssrn.3582908 (accessed April 22, 2020).

[B2] Barnett-Howell Z. Watson O. J. Mobarak A. M. (2021). The benefits and costs of social distancing in high- and low-income countries. *Trans. R. Soc. Trop. Med. Hyg.* 115 807–819. 10.1093/trstmh/traa140 33440007 PMC7928561

[B3] Barrios J. M. Hochberg Y. V. (2021). Risk perceptions and politics: Evidence from the COVID-19 pandemic. *J. Financ. Econ.* 142 862–879. 10.1016/j.jfineco.2021.05.039 34658487 PMC8502491

[B4] Bish A. Michie S. (2010). Demographic and attitudinal determinants of protective behaviours during a pandemic: A review. *Br. J. Health Psychol.* 15 797–824. 10.1348/135910710X485826 20109274 PMC7185452

[B5] Bruine de Bruin W. Bennett D. (2020). Relationships between initial COVID-19 risk perceptions and protective health behaviors: A national survey. *Am. J. Prev. Med.* 59 157–167. 10.1016/j.amepre.2020.05.001 32576418 PMC7242956

[B6] Bruine de Bruin W. Saw H.-W. Goldman D. P. (2020). Political polarization in US residents’. COVID-19 risk perceptions, policy preferences, and protective behaviors. *J. Risk Uncertain.* 61 177–194. 10.1007/s11166-020-09336-3 33223612 PMC7672261

[B7] Clinton J. Cohen J. Lapinski J. Trussler M. (2021). Partisan pandemic: How partisanship and public health concerns affect individuals’ social mobility during COVID-19. *Sci. Adv.* 7 eabd7204. 10.1126/sciadv.abd7204 33310734 PMC7787499

[B8] Deb P. Furceri D. Ostry J. D. Tawk N. (2022). The economic effects of COVID-19 containment measures. *Open Econ. Rev.* 33 1–32. 10.1007/s11079-021-09638-2

[B9] DiMatteo M. R. Lepper H. S. Croghan T. W. (2000). Depression is a risk factor for noncompliance with medical treatment: Meta-analysis of the effects of anxiety and depression on patient adherence. *Arch. Intern. Med.* 160 2101–2107. 10.1001/archinte.160.14.2101 10904452

[B10] Dryhurst S. Schneider C. R. Kerr J. Freeman A. L. J. Recchia G. Bles A. M. (2020). Risk perceptions of COVID-19 around the world. *J. Risk Res.* 23 994–1006. 10.1080/13669877.2020.1758193

[B11] Floyd D. L. Prentice-Dunn S. Rogers R. W. (2000). A meta-analysis of research on protection motivation theory. *J. Appl. Soc. Psychol.* 30 407–429. 10.1111/j.1559-1816.2000.tb02323.x

[B12] Freira L. Sartorio M. Boruchowicz C. Lopez Boo F. Navajas J. (2021). The interplay between partisanship, forecasted COVID-19 deaths, and support for preventive policies. *Humanit. Soc. Sci. Commun.* 8:192. 10.1057/s41599-021-00870-2

[B13] Furnham A. Boo H. C. (2011). A literature review of the anchoring effect. *J. Socio Econ.* 40 35–42. 10.1016/j.socec.2010.10.008

[B14] Grossman G. Kim S. Rexer J. M. Thirumurthy H. (2020). Political partisanship influences behavioral responses to governors’ recommendations for COVID-19 prevention in the United States. *Proc. Natl. Acad. Sci. U.S.A.* 7 24144–24153. 10.1073/pnas.2007835117 32934147 PMC7533884

[B15] Han Q. Zheng B. Cristea M. Agostini M. Bélanger J. J. Gützkow B. (2021). Trust in government regarding COVID-19 and its associations with preventive health behaviour and prosocial behaviour during the pandemic: a cross-sectional and longitudinal study. *Psychol. Med.* 26 1–11. 10.1017/S0033291721001306 33769242 PMC8144822

[B16] Kahan D. M. (2016). “The politically motivated reasoning paradigm, part 1: What politically motivated reasoning is and how to measure It,” in *Emerging trends in the social and behavioral sciences*, (Hoboken, NJ: John Wiley & Sons, Ltd), 1–16. 10.1002/9781118900772.etrds0417

[B17] Kroenke K. Spitzer R. L. Williams J. B. W. Löwe B. (2009). An ultra-brief screening scale for anxiety and depression: the PHQ-4. *Psychosomatics* 50 613–621. 10.1176/appi.psy.50.6.613 19996233

[B18] Liu S. Dobriban E. (2019). Ridge regression: Structure, cross-validation, and sketching. *arXiv* [Preprint] 10.48550/arXiv.1910.02373 35895330

[B19] Ning L. Niu J. Bi X. Yang C. Liu Z. Wu Q. (2020). The impacts of knowledge, risk perception, emotion and information on citizens’ protective behaviors during the outbreak of COVID-19: A cross-sectional study in China. *BMC Public Health* 20:1751. 10.1186/s12889-020-09892-y 33225934 PMC7681179

[B20] Pagliaro S. Sacchi S. Pacilli M. G. Brambilla M. Lionetti F. Bettache K. (2021). Trust predicts COVID-19 prescribed and discretionary behavioral intentions in 23 countries. *PLoS One* 16:e0248334. 10.1371/journal.pone.0248334 33690672 PMC7946319

[B21] Petherick A. Goldszmidt R. Andrade E. B. Furst R. Hale T. Pott A. (2021). A worldwide assessment of changes in adherence to COVID-19 protective behaviours and hypothesized pandemic fatigue. *Nat. Hum. Behav.* 5 1145–1160. 10.1038/s41562-021-01181-x 34345009

[B22] Roozenbeek J. Schneider C. R. Dryhurst S. Kerr J. Freeman A. L. J. Recchia G. (2020). Susceptibility to misinformation about COVID-19 around the world. *R. Soc. Open Sci.* 7:201199. 10.1098/rsos.201199 33204475 PMC7657933

[B23] Schneider C. R. Dryhurst S. Kerr J. Freeman A. L. J. Recchia G. Spiegelhalter D. (2021). COVID-19 risk perception: a longitudinal analysis of its predictors and associations with health protective behaviours in the United Kingdom. *J. Risk Res.* 24 294–313. 10.1080/13669877.2021.1890637

[B24] Senay I. Kaphingst K. A. (2009). Anchoring-and-adjustment bias in communication of disease risk. *Med. Decis. Mak. Int. J. Soc. Med. Decis. Mak.* 29 193–201. 10.1177/0272989X08327395 19279297 PMC2668745

[B25] Sheeran P. Harris P. R. Epton T. (2014). Does heightening risk appraisals change people’s intentions and behavior? A meta-analysis of experimental studies. *Psychol. Bull.* 140 511–543. 10.1037/a0033065 23731175

[B26] Slovic P. Peters E. Finucane M. L. MacGregor D. G. (2005). Affect, risk, and decision making. *Health Psychol.* 24 S35–S40. 10.1037/0278-6133.24.4.S35 16045417

[B27] Torrente F. Yoris A. Low D. M. Lopez P. Bekinschtein P. Manes F. (2021). Sooner than you think: A very early affective reaction to the COVID-19 pandemic and quarantine in Argentina. *J. Affect. Disord.* 282 495–503. 10.1016/j.jad.2020.12.124 33422827 PMC8529255

[B28] Torrente F. Yoris A. Low D. Lopez P. Bekinschtein P. Vázquez G. H. (2022). Psychological symptoms, mental fatigue and behavioural adherence after 72 continuous days of strict lockdown during the COVID-19 pandemic in Argentina. *BJPsych Open* 8:e10. 10.1192/bjo.2021.1065 34931146 PMC8668400

[B29] Tversky A. Kahneman D. (1974). Judgment under uncertainty: Heuristics and biases. *Science* 185 1124–1131. 10.1126/science.185.4157.1124 17835457

[B30] van der Linden S. (2015). The social-psychological determinants of climate change risk perceptions: Towards a comprehensive model. *J. Environ. Psychol.* 41 112–124. 10.1016/j.jenvp.2014.11.012

[B31] Wing R. R. Phelan S. Tate D. (2002). The role of adherence in mediating the relationship between depression and health outcomes. *J. Psychosom. Res.* 53 877–881. 10.1016/S0022-3999(02)00315-X 12377297

