# Supplementary material for: Risk perception, but also political orientation, modulate behavioral response to COVID-19: A randomized survey experiment
Source: Front Psychol. 2022 Aug 17;13:900684. doi: 10.3389/fpsyg.2022.900684 (PMC9428706; doi:10.3389/fpsyg.2022.900684)

Supplementary Material

Risk perception, but also political orientation, modulate behavioral response to COVID-19: a randomized survey experiment.

Fernando Torrente [1,2*], Daniel Low [3], Adrian Yoris[1,2]

[1] Institute of Neuroscience and Public Policy, INECO Foundation, Buenos Aires, Argentina

[2] Institute of Cognitive and Translational Neurosciences (CONICET-INECO Foundation-Favaloro University), Buenos Aires, Argentina

[3] Program in Speech and Hearing Bioscience and Technology, Harvard Medical School & MIT, USA

*Corresponding author. Email: ftorrente@ineco.org.ar

This PDF file includes:

Tables S1 to S9

Figures S1, S2 & S3

Table S1. Variables related with risk perception, health protective behaviors and appraisal of the health context

Variable	Question	Answers (scoring)
Personal Risk perception (Severity)	'If you were get infected with COVID-19, do you think the disease in your case would be…'.	'Very serious' (4)<br>'Serious' (3)<br>'Moderately serious' (2)<br>'Slightly or not serious' (1)<br>'I don't know' (NS)
Personal Risk perception (Susceptibility)	'To what extent do you feel vulnerable to being infected with COVID-19…	'Very vulnerable' (4)<br>'Quite a bit' (3)<br>'A little' (2)<br>'Not at all vulnerable' (1)<br>'I don't know' (NS)
Personal Risk perception (Fear of Covid-19)	'Speaking of the fear of the COVID-19, personally...'.	'I'm afraid of it, and that's why I leave home only for the indispensable' (4)<br>'I'm afraid of it, but I continue my activities with the necessary care' (3)<br>'I used to be afraid of it, but not anymore' (2)<br>'I was never afraid of it' (1)<br>'I don't know' (NS)
Protective health behaviors (Use of face mask)	'When you leave your home, in your day to day, do you use a face mask?'	'Always' (4)<br>'Most of the time' (3)<br>'Sometimes' (2)<br>'Never' (1)<br>'I don't know' (NS)
Protective health behaviors (Physical distancing)	'In your daily life, do you maintain physical distance from other people outside your home?'	'Always' (4)<br>'Most of the time' (3)<br>'Sometimes' (2)<br>'Never' (1)<br>'I don't know' (NS)
Protective health behaviors (Avoidance of enclosed spaces)	'Regarding closed spaces such as restaurants, shopping malls or theaters? You...'.	'Does not go indoors' (4)<br>'Goes only for short periods (up to 15 minutes)' (3)<br>'Goes only if they are adequately ventilated' (2)<br>'Goes indoors sometimes'<br>'Goes indoors without inconvenience' (1)<br>'I don't know' (NS)
Appraisal of health context (current)	'What do you think about the COVID-19 situation regarding the number of cases in our country, the occupation of intensive care beds, and the	'Very serious' (4)<br>Fairly serious' (3)<br>'A little serious' (2)<br>'Not serious at all' (1)<br>'I don't know' (NS)

	mortality due to the virus? Do you think that the current health situation is...'.	
Appraisal of health context (future)	'In the next three months the health situation due to the COVID-19 in Argentina (number of cases, bed occupancy and mortality) is going to be...'.	'Much worse than now' (5)<br>'Worse than now' (4)<br>'Same as now' (3)<br>'Better than now' (2)<br>'Much better than now' (1)<br>'I don't know' (NS)
Support for restriction measures	'If the cases of coronavirus infections in the country increased substantially, you believe that the most convenient thing would be...'.	'Strictly close everything again, like in March last year' (5)<br>'Restrict only activities in enclosed spaces (including schools and work activities)' (4)<br>'Restrict activities in enclosed spaces, but not schools and work activities' (3)<br>'Continue with the current measures' (2)<br>'Allow all activities' (1)<br>'I don't know' (NS)

Note: NS: Not scored.

Table S2. Content of randomized messages

Type of Message	Content	Answers
Emotional risk enhancement message	"Taking into account that the new strains of the virus can be much more contagious and lethal than the previous, would you be willing to accept restrictions on your normal life?"	'Yes, as many restrictions as are necessary' (2)<br>'Only a few more restrictions than those that already exist' (1)<br>'I am only willing to maintain the restrictions that already exist' (0)<br>'I would not be willing to accept any restrictions to my normal life' (0)<br>'I don't know' (NS)
Cognitive risk enhancement message	'Taking into account that the number of COVID-19 cases in some areas of the country are close to the values of the 2020 peak and are likely to exceed it, would you be willing to accept restrictions on your normal life?'	'Yes, as many restrictions as are necessary' (2)<br>'Only a few more restrictions than those that already exist' (1)<br>'I am only willing to maintain the restrictions that already exist' (0)<br>'I would not be willing to accept any restrictions to my normal life' (0)<br>'I don't know' (NS)
Pro-social message	'Taking into account that there are still many older people and risk groups without vaccination, would you be willing to accept restrictions on your normal life?'	'Yes, as many restrictions as are necessary' (2)<br>'Only a few more restrictions than those that already exist' (1)<br>'I am only willing to maintain the restrictions that already exist' (0)<br>'I would not be willing to accept any restrictions to my normal life' (0)<br>'I don't know' (NS)

Note: NS: Not scored.

Table S3. Appraisal of current and future health context

		Age				
		Up to 29	30 to 49	50 to 65	66+	Total
**Current**						
Very Serious	n	86.00	204.00	268.00	203.00	761.00
	%	35.83	27.24	24.17	25.50	26.30
Serious	n	96.00	357.00	557.00	389.00	1.399.00
	%	40.00	47.66	50.23	48.87	48.34
Little serious	n	28.00	110.00	175.00	110.00	423.00
	%	11.67	14.69	15.78	13.82	14.62
Not at all serious	n	17.00	28.00	36.00	24.00	105.00
	%	7.08	3.74	3.25	3.02	3.63
I don’t know	n	13.00	50.00	73.00	70.00	206.00
	%	5.42	6.68	6.58	8.79	7.12
**Future (3 months)**						
Much worst	n	79.00	253.00	317.00	184.00	833.00
	%	32.92	33.78	28.58	23.12	28.78
Worst	n	77.00	250.00	379.00	256.00	962.00
	%	32.08	33.38	34.18	32.16	33.24
No change	n	41.00	125.00	228.00	197.00	591.00
	%	17.08	16.69	20.56	24.75	20.42
Better	n	20.00	54.00	82.00	86.00	242.00
	%	8.33	7.21	7.39	10.80	8.36
Much better	n	9.00	13.00	15.00	12.00	49.00
	%	3.75	1.74	1.35	1.51	1.69
I don’t know	n	14.00	54.00	88.00	61.00	217.00
	%	5.83	7.21	7.94	7.66	7.50

Table S4. Personal Risk perception of COVID-19 (n = 2894)

		Age				
		**Up to 29**	**30 to 49**	**50 to 65**	**66+**	**Total**
**Perceived severity**						
Very severe	n	39	119	251	222	631
	%	16.25	15.89	22.63	27.89	21.80
Severe	n	29	138	229	184	580
	%	12.08	18.42	20.65	23.12	20.04
Moderately Severe	n	57	169	266	145	637
	%	23.75	22.56	23.99	18.22	22.01
Mildly or not severe	n	94	231	259	172	756
	%	39.17	30.84	23.35	21.61	26.12
I don’t know	n	21	92	104	73	290
	%	8.75	12.28	9.38	9.17	10.02
**Perceived susceptibility**						
Very much	n	34	117	138	105	394
	%	14.17	15.62	12.44	13.19	13.61
Quite	n	53	219	306	195	773
	%	22.08	29.24	27.59	24.50	26.71
A little	n	89	256	413	275	1033
	%	37.08	34.18	37.24	34.55	35.69
Nothing at all	n	41	68	111	116	336
	%	17.08	9.08	10.01	14.57	11.61
I don't know	n	23	89	141	105	358
	%	9.58	11.88	12.71	13.19	12.37
**Fear of COVID-19**						
Never feared	n	53	96	142	99	390
	%	22.08	12.82	12.80	12.44	13.48
Before, but no longer	n	25	67	47	42	181
	%	10.42	8.95	4.24	5.28	6.25
'I'm afraid of it, but I continue my activities with the necessary care'	n	103	383	509	313	1308
	%	42.92	51.13	45.90	39.32	45.20
I´m afraid of it, I only go out for the essential	n	52	185	393	326	956
	%	21.67	24.70	35.44	40.95	33.03
I don't know	n	7	18	18	16	59
	%	2.92	2.40	1.62	2.01	2.04

Table S5. Distribution of participants in high and low personal risk categories across age groups

		Age				
		Up to 29	30 to 49	50 to 65	66+	Total*
**Perceived Severity[1]**						
Low	n	151	400	525	317	1393
	%	68.950 %	60.883 %	52.239 %	43.845 %	53.495 %
High	n	68	257	480	406	1211
	%	31.050 %	39.117 %	47.761 %	56.155 %	46.505 %
**Perceived Susceptibility[2]**						
Low	n	130	324	524	391	1369
	%	59.908 %	49.091 %	54.132 %	56.585 %	53.983 %
High	n	87	336	444	300	1167
	%	40.092 %	50.909 %	45.868 %	43.415 %	46.017 %
**Fear of COVID-19[3]**						
Low	n	78	163	189	140	570
	%	33.476 %	22.298 %	17.324 %	17.972 %	20.113 %
High	n	155	568	902	639	2264
	%	66.524 %	77.702 %	82.676 %	82.028 %	79.887 %

1. $X^2 = 63.140$, $p < .001$.
2. $X^2 = 11.316$, $p = .01$.
3. $X^2 = 35.575$, $p < .001$.

*= The Total column includes all participants who answered anything other than "I don't know." Therefore, the percentages are different from those presented in Table S4.

Table S6. Frequencies of specific health protective behaviors

		Age				
		Up to 29	30 to 49	50 to 65	66+	Total
**Mask wearing**						
Always	n	173	593	940	704	2.410
	%	72.08	79.17	84.76	88.44	83.28
Most of the time	n	35	108	112	59	314
	%	14.58	14.42	10.10	7.41	10.85
Sometimes	n	22	39	47	23	131
	%	9.17	5.21	4.24	2.89	4.53
Never	n	9	9	9	7	34
	%	3.75	1.20	0.81	0.88	1.17
Do not know	n	1	0	1	3	5
	%	0.48	0	0.09	0.38	0.17
**Distancing**						
Always	n	124	456	786	602	1.968
	%	51.67	60.88	70.87	75.63	68.00
Most of the time	n	68	224	259	155	706
	%	28.33	29.91	23.35	19.47	24.39
Sometimes	n	34	56	51	26	167
	%	14.17	7.48	4.60	3.27	5.77
Never	n	12	11	10	11	44
	%	5.00	1.47	0.90	1.38	1.52
Do not know	n	2	2	3	2	9
	%	0.83	0.27	0.27	0.25	0.31
**Avoid closed spaces**						
Do not go	n	117	430	753	589	1.889
	%	48.75	57.41	67.90	73.99	65.27
For short periods	n	24	79	95	67	265
	%	10.00	10.55	8.57	8.42	9.16
Only ventilated	n	31	103	133	79	346
	%	12.92	13.75	11.99	9.92	11.96
Sometimes	n	43	96	85	32	256
	%	17.92	12.82	7.66	4.02	8.85
Without restrictions	n	21	27	21	9	78
	%	8.75	3.60	1.89	1.13	2.69
Do not know	n	4	14	22	20	60
	%	1.67	1.87	1.98	2.51	2.07

Table S7. Predictive performance and coefficients for Ridge regression models

	Support for restrictions OOS R^2=0.15 (0.02)		Personal risk index OOS R^2=0.21 (0.03)		Protective health behaviors index OOS R^2=0.17 (0.01)	
	Covariate	Coef.	Covariate	Coef.	Covariate	Coef.
1	Politically opposed	-0.32***	Health Context Appraisal (current)	0.38***	Age groups	0.29***
2	Personal Risk Index	0.15***	Psychological distress (PHQ4)	0.34***	Personal Risk Index	0.21***
3	Protective Health Behaviors Index	0.14***	Health Context Appraisal (future)	0.3***	Health Context Appraisal (current)	0.19***
4	Health Context Appraisal (current)	0.12***	Protective Health Behaviors Index	0.27***	Support for restrictions	0.17***
5	Health Context Appraisal (future)	0.09***	Support for restrictions	0.25***	Region	-0.12***
6	Vaccinated	0.06**	Politically opposed	-0.25***	Female (binary)	0.11***
7	Education level	0.05	Age groups	0.25***	Psychological distress (PHQ4)	-0.1***
8	Age groups	-0.05*	Region	-0.08*	Politically opposed	-0.1***
9	Psychological distress (PHQ4)	-0.03	Female (binary)	0.04	Health Context Appraisal (future)	0.1***
10	SES	-0.02	Education level	0.03	Vaccinated	0.08***
11	Region	-0.01	Vaccinated	-0.03	Past Covid19 diagnosis	-0.04
12	Past Covid19 diagnosis	0	Past Covid19 diagnosis	-0.03	SES	-0.04
13	Female (binary)	0	SES	0.01	Education level	-0.01

Note: OOS R2 is out-of-sample prediction mean (standard deviation) across 5 test sets using 5-fold cross-validation. Variables are ranked by their absolute coefficient values. The standardized coefficients represent how many standard deviations a dependent variable will change per standard deviation increase in the covariate (e.g.. 1 s.d. increase in being politically opposed is associated with 0.32 decrease in Support for restrictions, if all other covariates are fixed). Stronger associations have higher absolute coefficient values. Positive coefficients make it more likely that the dependent variable will increase; negative coefficients make it more likely that the dependent variable will decrease. Coefficients closer to zero are not associated to the dependent variables. OOS: out-of-sample; Coef.: coefficient; PHQ-4: Patient Health Questionnaire-4; SES: socio-economical status. *** = P-value <=0.001; ** = P-value <=0.01; * = P-value <=0.05.

Table S8. Ordinal logistic regression of the effect of type of message on restrictions acceptance

a. *Model fitting information*

Model	-2 log likelihood	Chi-square	df	Sig.
Only intersection	52.249			
Final	43.211	9.038	2	.011

b. *Test of paralell lines*

Model	-2 log likelihood	Chi-square	df	Sig.
Null	43.211			
General	41.928	1.283	2	.527

c. *Goodness-of-fit*

	Chi-square	df	Sig.
Pearson	1.280	2	.527
Deviance	1.283	2	.527

d. *Parameter estimates*

	B	SE	Wald	gl	Sig.	OR	Lower	Upper
Emotional-risk message	.189	.0846	4.999	1	.025	1.208	1.024	1.426
Cognitive-risk message	.246	.0864	8.104	1	.004	1.279	1.080	1.515
Pro-social message	0[a]	.	.	.	.	1	.	.

Link function: Logit.

[a.] This parameter is set to zero because it is redundant.

Table S9. Ordinal clog-log regression of the effect of type of message and political orientation on restrictions acceptance

a. *Model fitting information*

Model	-2 log likelihood	Chi-square	df	Sig.
Only intersection	539.722			
Final	85.162	454.560	3	<.001

b. Test of paralell lines

Model	-2 log likelihood	Chi-square	df	Sig.
Null	85.162			
General	83.306	1.856	3	.603

c. Goodness-of-fit

	Chi-square	df	Sig.
Pearson	12.334	7	.090
Deviance	12.141	7	.096

d. Parameter estimates

	B	SE	Wald	gl	Sig.
Emotional-risk message	.187	.062	9.008	1	.003
Cognitive-risk message	.242	.064	14.270	1	<.001
Pro-social message	0[a]	.	.	0	.
Government supporters	1.212	.063	369.267	1	<.001
Government opposers	0[a]	.	.	0	.

Link function: complementary log–log.

[a.] This parameter is set to zero because it is redundant.

Figure S1. Dimensions of perceived risk of COVID-19

a. Perceived Severity of COVID-19 infection

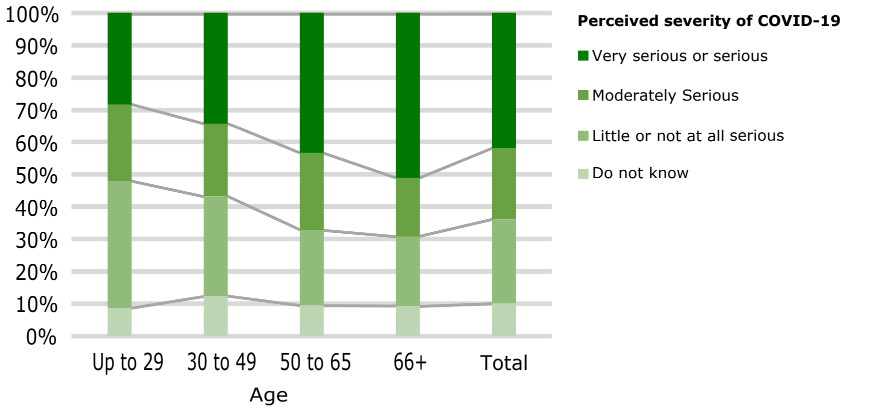

b. Perceived Susceptibility to COVID-19 infection

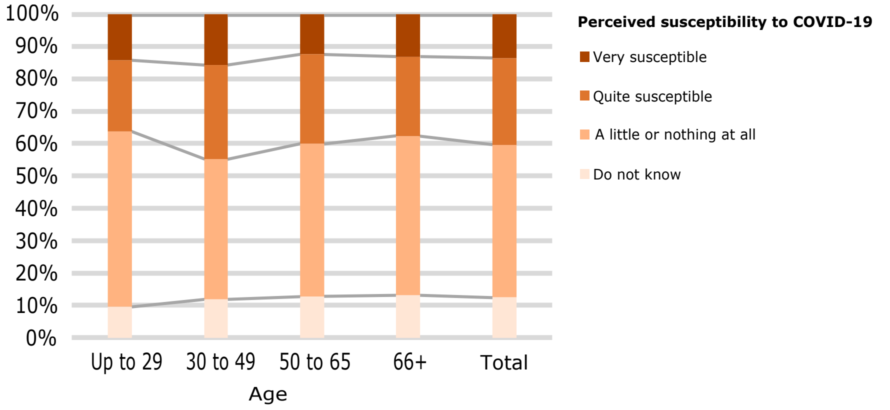

c. Fear to COVID-19

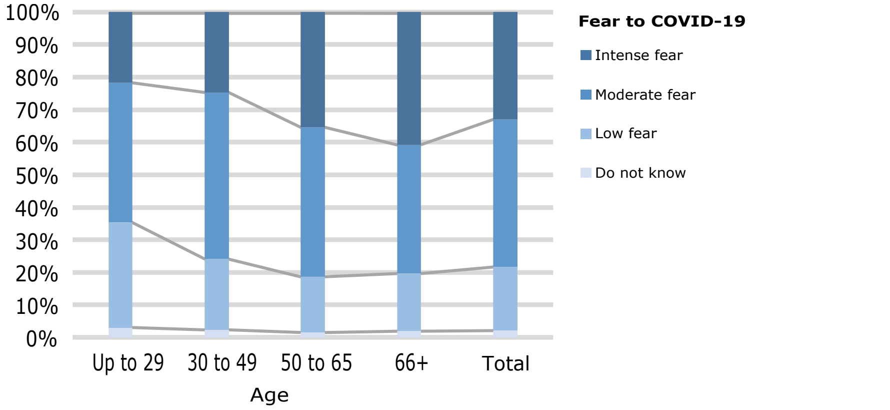

Figure S2. Distribution and medians of participant's' responses to the three types of messages in the total sample (A), pro-government participants (B), and opponents to the government (C). P-values shown correspond to post-hoc pairwise comparisons (Dunn test with Holm correction) after Kruskal-Wallis test.

A

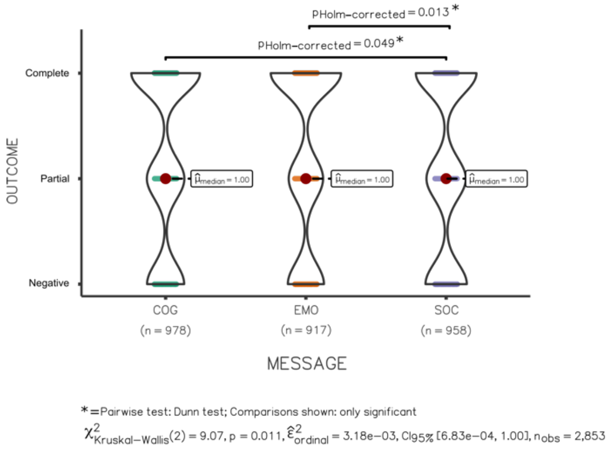

B

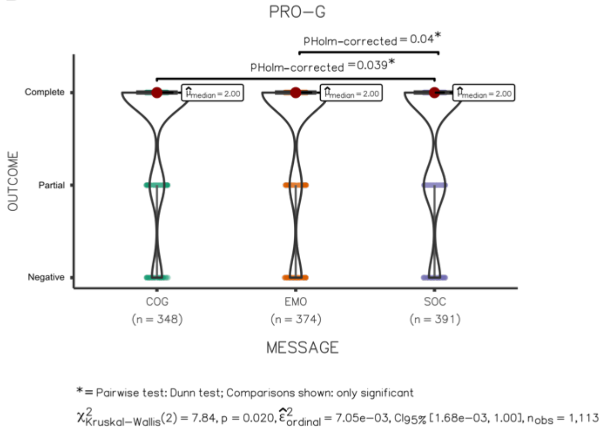

C

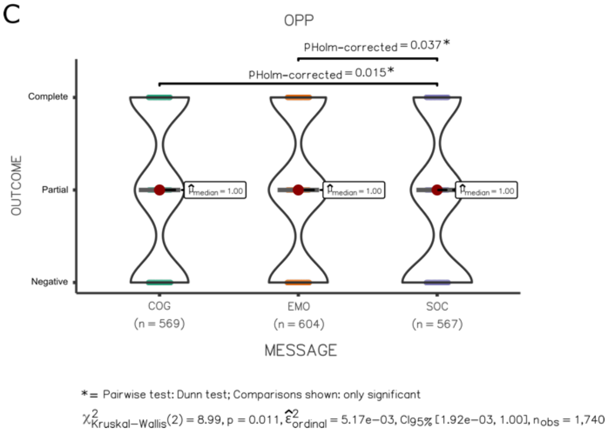

Figure S3. DAG Diagram of main variables of interest

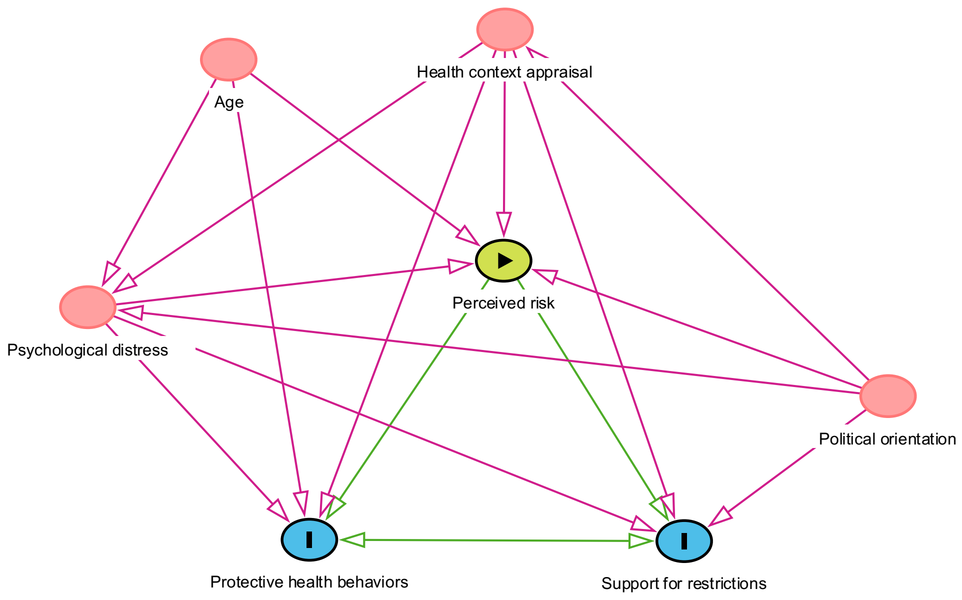

Supplement: Supplementary file 1 [file Data_Sheet_1.PDF]
